# The effects of a mixture of small peptide chelating minerals and inorganic minerals on the production performance and tissue deposition of broiler chickens

**DOI:** 10.3389/fvets.2024.1380911

**Published:** 2024-04-19

**Authors:** Xiaofeng Han, Jing Kong, Chaojun Zheng, Xia Yan, Ting Qiu, Zhiyong Chen, Huihua Zhang

**Affiliations:** ^1^School of Life Science and Engineering, Foshan University, Foshan, China; ^2^Wen’s Foodstuffs Group Co., Ltd., Yunfu, China; ^3^Guangdong Academy of Agricultural Sciences, Guangzhou, China; ^4^Foshan Guangmuxing Feed Co., Ltd., Foshan, China

**Keywords:** inorganic trace mineral, small peptide chelate mineral, growth performance, deposition of trace elements, antioxidant status

## Abstract

Due to the limited bioavailability of inorganic trace minerals, their utilization in poultry production has led to problems such as environmental contamination and inefficient resource utilization. It was investigated whether replacing inorganic trace minerals (ITM) with a blend of organic small peptide-chelated trace minerals (MIX) would improve production performance, selected biochemical parameters, antioxidant capacity, mineral deposition in liver, heart, and tibia, as well as mineral content in feces of broilers. A total of 432 healthy 21-day-old 817 broilers were randomly divided into 4 groups with 6 replicates per group and 18 chickens per replicate. The control group received a basal diet supplemented with 1,000 mg/kg of inorganic trace minerals as sulfate. The experimental groups received basal diets supplemented with 200, 400, and 600 mg/kg of mixed trace mineral elements (50% sulfate +50% small peptide-chelate) for a trial period of 30 days, divided into two stages: 21–35 days and 36–50 days. The results indicate that on the 50th day, compared with the 1,000 mg/kg ITM group, the levels of serum cholesterol, urea nitrogen, and malondialdehyde in the 200, 400, and 600 mg/kg MIX groups decreased (*p* < 0.01), while the levels of serum glutathione peroxidase in the 200, 400, and 600 mg/kg MIX groups increased (*p* < 0.05). Compared to the ITM group, the addition of organic small peptide chelated trace minerals mixed with inorganic trace minerals can reduce the levels of zinc and manganese in feces (*p* < 0.01). Furthermore, the iron content in the heart and tibia of the 600 mg/kg MIX group also significantly decreased (*p* < 0.05). There were no differences in growth performance and slaughter performance among the groups (*p* > 0.05). This study shows that replacing inorganic minerals with low-dose MIX (200, 400, and 600 mg/kg) can reduce the levels of zinc and manganese in feces, with no negative impact on growth and slaughter performance.

## Introduction

Due to high protein needs and demand of humans, poultry particularly broiler meat is getting popularity due to its high nutritive values in terms of proteins and other bioactive compounds including vitamins, fatty acids and antioxidants which are very beneficial as far as human health is concerned (1) and essential nutrients like zinc, copper, iron and manganese play pivotal role in the efficient growth of broiler chicks (2). Directly or indirectly, these elements are involved in animal physiology and biochemistry (3). The deficiency of these trace elements can result in non-specific symptoms, including reduced feed intake, delayed growth, and even mortality (4). Typically, inorganic salts are preferred as the primary choice for supplementing trace elements in feed, while the bioavailability of trace minerals in inorganic salt form is generally low (5). Trace elements in feed accumulate in the environment with excreta, which can lead to various issues in the long term, such as soil pollution, toxicity, decreased crop yield, and resource wastage (6). The impact of this issue extends beyond the sustainability of the livestock industry and also affects the future availability of arable land. Consequently, it is imperative to explore mineral alternatives with enhanced bioavailability.

Organic trace elements prepared by chelation with amino acids, proteins, and other organic substances have advantages such as high biological availability, high biological efficacy, and good stability. Consequently, they are increasingly replacing inorganic trace elements (7). The enhanced bioavailability of organic trace minerals originates from their absorption through amino acid or peptide transport systems. (8). By substituting low levels of amino acid chelates for traditional inorganic salts in animal feed, it is possible to significantly reduce the content of trace elements in feces without affecting the growth performance of broiler chickens, thereby alleviating issues of resource waste and environmental pollution.

Research has shown that supplementing 4 mg/kg of copper from organic sources may be sufficient to maintain normal growth of broiler chickens (9). Compared to the use of sulfate form at the recommended dosage, a significant reduction of copper in the form of glycine chelate compounds does not lead to degeneration of the structure and morphological characteristics of chicken tibias (10). Therefore, copper plays an important role in skeletal development, so the use of more easily absorbable chelated forms of copper may improve the growth and development of the skeletal system in broiler chickens. Gheisari et al. (11) suggested adding 40 mg/kg of manganese from organic sources in the diet as a substitute for 100 mg/kg of manganese oxide to maintain the production performance of broiler chickens. Xiao et al. (12), demonstrated that manganese-amino acid chelates, as organic sources, improved the quality parameters of eggshell in older laying hens compared to inorganic manganese sulfate. Organic forms of manganese are more efficient than inorganic forms (manganese sulfate), and the use of methionine as a ligand promotes the absorption of manganese more effectively than glycine (13). In a study by Kwiecień et al. (9) on broiler chickens from 1 to 42 days of age, organic iron and inorganic iron were supplemented in the diet. The results showed that adding iron in the form of glycine chelate at 25% or 50% of the amount of inorganic iron supplementation was sufficient to maintain the production performance and health of broiler chickens. Brooks et al. (14) described the positive effects of adding zinc propionate in diets with reduced levels of zinc (less than 22 mg/kg) which include increased feed intake, body weight, and tibial zinc concentration. Studies have shown that the addition of organic forms of zinc significantly increases the concentration of this trace element in the plasma, which can be explained by the higher bioavailability of Zn-Met (15).

In recent years, environmental issues have become increasingly severe, and people are paying more attention to alleviating heavy metal pollution. Previous studies have shown that low-dose peptide-chelated minerals have advantages over inorganic trace mineral supplements because organic minerals have higher biological availability. Research has found that a mixture of small peptide chelated minerals used to completely replace inorganic minerals in feed does not have a negative impact on the production performance of broiler chickens. On the contrary, it can enhance serum biochemical markers and liver antioxidant activity, while reducing the mineral content in feces (16). Thus, this study aims to investigate the effects of different concentrations of peptide-chelated minerals mixed with inorganic minerals in broiler feed on growth performance, serum biochemical markers, antioxidant capacity, tissue deposition, and fecal excretion. The purpose of this study is to provide empirical evidence for the practical application of peptide-chelated minerals in broiler production.

## Materials and methods

### Animals, group formation and diets

This experiment followed the principle of similar body weight and selected 432 healthy 21-day-old 817 fast-growing broiler chickens. In each group, six replicates were conducted, each containing 18 chicks. All the experimental chickens were raised in 66 cm x 66 cm x 43 cm wire cages and had free access to feed. They were fed on a schedule, with the amount of feed remaining in the trough used as a measure, and water was provided through automatic drinkers. Deworming and immunization were carried out according to routine procedures and methods. The management of chicken feeding was based on standard farm practices (no other commercial feed additives and supplements added to our feed). Feed and clean water were provided to all the birds during the study. Animal research ethics committee approval was obtained from Foshan University. The feeding was divided into two phases (21–35 days and 36–50 days), and the entire experiment lasted for 30 days. The basal diet was a corn-soybean meal-based diet formulated according to the National Research Council (NRC 1998) and adjusted according to the Chinese agricultural industry standard (NY/T33-2004, chicken feed standard). The composition and nutrient levels of the diet are shown in Table 1.

**Table 1:** Basic diet’s ingredients and nutritional composition.

Item	STARTER 21-35d	FINISHER 36-50d
Ingredients		
Corn	58.49	57.66
Soybean meal	29.08	27.21
Corn protein powder	5.00	5.00
Stone Powder	1.36	1.22
DCP	1.00	1.04
L-lysine sulfate	0.49	0.39
DL-Methionine	0.27	0.21
NaCl	0.32	0.32
L-Threonine	0.10	0.06
Choline chloride (50%)	0.08	0.08
Vitamin and mineral premix^a^	0.35	0.35
Phytase	0.01	0.01
Sodium humate	0.10	0.15
Lard	3.35	6.30
Calculated nutrient level		
ME (MJ/kg)	12.80	13.52
CP	20.62	19.61
EE	6.04	8.87
*CF*	2.42	2.33
Ca	0.85	0.8
TP	0.55	0.54
Lys	1.34	1.21
Met	0.58	0.52
Met + Cys	0.86	0.78
Thr	0.86	0.79

a Supplied per kilogram of diet: vitaminn A 12,000 IU vitamin D3 3,000 IU, vita-min E 101 IU, vitamin K 32 mg, vitamin B11 mg, vitamin B23 mg, vitamin B62 mg, vit-amin B12 0.01 mg, pantothenate 4 mg, nicotinic acid 20 mg, folic acid 0.5 mg, biotin 0.05 mg.

Trace minerals are supplemented regarding the experimental design and added to the premix.

According to Table 2, the dosages of minerals supplemented to the four dietary treatments differ. The control group was supplemented with 1,000 mg/kg of sulfate trace mineral in the basal diet. The experimental groups were supplemented with mixed trace mineral elements (50% sulfate +50% small peptide chelates) at levels of 200, 400, and 600 mg/kg in the basal diet, respectively. The trace minerals were provided by Xing tengke Biotechnology Co., Ltd. (Guangdong, China).

**Table 2:** Experimental treatment of broiler diets and supply of each trace element (mg/kg).

	Inorganic^a^	Organic and inorganic mixed^b^		
Item	1,000 mg/kg ITM	200 mg/kg MIX	400 mg/kg MIX	600 mg/kg MIX
Cu	6	1.1	2.2	3.3
Fe	30	6	12	18
Zn	50	7	14	21
Mn	60	12	24	36

The 1,000 mg/kg ITM group was supplemented with 1,000 mg/kg of sulfate trace minerals in the basal diet; The 200 mg/kg MIX group was supplemented with mixed trace mineral elements (50% sulfate + 50% small peptide chelates); The 400 mg/kg MIX group was supplemented with mixed trace mineral elements (50% sulfate + 50% small peptide chelates); The 600 mg/kg MIX group was supplemented with mixed trace mineral elements (50% sulfate + 50% small pep-tide chelates).

a Inorganic trace elements copper, iron, zinc, and manganese added to the diet are sulfates.

b Organic and inorganic mixed elements copper, iron, zinc, and manganese added to the diet are small peptide chelates.

#### Feed intake and growth performance

Daily feed intake (FI) was monitored. Daily feed intake was calculated by subtracting the feed refused on previous day from the feed offered next day while feed conversion rations was calculated by dividing the feed intake of the birds (grams) with the weight gain of the bird (gram) as described by Al-Hoshani et al. (17). Body weight was measured before morning feeding on day 35 and day 50, respectively. Average daily feed intake (ADFI), average daily gain (ADG), and feed conversion ratio (FCR) were calculated for each growth stage (21–35, 35–50, and 21–50 days).

### Slaughter performance indicators

In the 50 day period, each group selects 6 chickens (each repetition resulted in the selection of one chicken). Before slaughter, chickens fast for 12 h and are then weighed. Their slaughter performance is evaluated through measures such as bleeding, feather removal, dissection, and measurement of various indicators. The slaughter indicators are determined according to the Chinese standard NY/T823-2004 “Naming and Metrological Statistic of Performance in Poultry Production”:

$$\mathrm{Slaughter}\;\mathrm{rate}\;\left(\%\right)=\mathrm{carcass}\;\mathrm{weight}/\mathrm{live}\;\mathrm{weight}\;x\;100.$$

$$\begin{array}{l} \mathrm{Leg}\;\mathrm{muscle}\;\mathrm{rate}\;\left(\%\right)=\mathrm{weight}\;\mathrm{of}\;\mathrm{leg}\;\mathrm{muscle}\;\mathrm{on}\;\mathrm{both}\;\mathrm{sides}/\\ \;\mathrm{total}\;\mathrm{eviscerated}\;\mathrm{weight}\;x\;100. \end{array}$$

$$\begin{array}{l} \mathrm{Breast}\;\mathrm{muscle}\;\mathrm{rate}\;\left(\%\right)=\mathrm{weight}\;\mathrm{of}\;\mathrm{breast}\;\mathrm{muscle}\;\mathrm{on}\;\mathrm{both}\;\mathrm{sides}/\\ \;\mathrm{total}\;\mathrm{eviscerated}\;\mathrm{weight}\;x\;100. \end{array}$$

$$\begin{array}{l} \mathrm{Abdominal}\;\mathrm{fat}\;\mathrm{rate}\;\left(\%\right)=\mathrm{weight}\;\mathrm{of}\;\mathrm{abdominal}\;\mathrm{fat}/\\ \left(\mathrm{weight}\;\mathrm{of}\;\mathrm{abdominal}\;\mathrm{fat}+\mathrm{total}\;\mathrm{eviscerated}\;\mathrm{weight}\right)\;x\;100. \end{array}$$

### Serum biochemical indicators

On days 35 and 50, each group selects 6 chickens (each repetition resulted in the selection of one chicken). After a 12-h fasting period, brachial vein blood samples were collected and allowed to stand for 2 h. The blood samples were centrifuged at 4°C and 3,000 rpm for 15 min, and the serum concentrations of albumin (ALB), alkaline phosphatase (AKP), total protein (TP), total cholesterol (T-CHO), and urea nitrogen (BUN) were measured using reagent kits from the Nanjing Biotechnology Research Institute in Jiangsu, China.

### Antioxidant indicators

On days 35 and 50, each group selects 6 chickens (each repetition resulted in the selection of one chicken). After blood collection, the chicks were euthanized by dis-locating their cervical vertebrae and blood was drained. We immediately removed the liver, weighed it, and stored it in liquid nitrogen to determine its antioxidant proper-ties. The liver sample was mixed with sterile, cold physiological saline (0.9%) (9:1) based on its weight, and then homogenized to obtain a tissue-free mass. The homogenate was centrifuged at 2500 × g for 10 min at 4°C. Antioxidant indicators such as catalase (CAT), malondialdehyde (MDA), total antioxidant capacity (T-AOC), glutathione peroxidase (GSH-Px), and copper-zinc superoxide dismutase (Cu/Zn-SOD) concentrations were analyzed in the liver and serum according to the instructions of the reagent kits from the Nanjing Biotechnology Research Institute in Jiangsu, China.

### Tissue and excreta trace elements

Tissue collection: Selected broilers with similar body weight were euthanized by cervical bleeding for liver antioxidant index determination. Samples were collected, and the remaining liver tissues were tightly wrapped in aluminum foil and rapidly stored in liquid nitrogen. The intact left leg muscles were stripped, and the intact tibias were preserved at low temperature. The collection work was completed and stored at-20°C.

Collection of chicken feces: Two experimental broilers from each replicate were pre-adapted in metabolic cages for 3 days with normal diet and water. Fecal samples were collected 3 days before the end of each experimental phase and stored at-20°C in the refrigerator.

Processing of liver, and heart: Removed excess fat and connective tissue from the samples, rinse them with deionized water, and used absorbent paper to dry the surface. Cut the large pieces of tissue into small fragments, grinded them into homogeneous samples using a mortar and pestle, and finally store them in polyethylene sample bags.

Processing of tibia: Removed the muscle tissue and took the intact left tibia. After marking the tibia, boil it in deionized water for about 15 min to removed surface soft tissue and cartilage. Transfer it to a constant temperature drying oven at 50 degrees Celsius for approximately 24 h. Subsequently, use petroleum ether for de-greasing for 48 h and then dry it. After drying, finely grind the defatted tibia using a stainless steel grinder, place it in a crucible, carbonize it smokelessly in an electric furnace, transfer it to a muffle furnace, and set it at 550 degrees Celsius for 16 h until it turns ash white. Stored it in a dry environment.

Processing of feces: Collected feces 3 days before the end of the 50-day experimental period, dry them in a drying oven for approximately 48 h at a temperature of 65 degrees Celsius. Once feces are dried, let them sit indoors for 24 h to regain moisture. Crush and mix the feces from each replicated chicken using a stainless steel blade grinder to obtain homogeneous fecal samples. Finally, sieve the fecal samples (0.5 mm) and store them in polyethylene sample bags. Trace elements in liver, pectoral muscle, heart, tibia ash, and feces (Zn, Fe, Mn, Cu) were determined by flame atomic absorption spectrometry. The determination of zinc, iron, manganese, and copper referred to the Chinese standards GB 5009.14–2017, GB 5009.90–2016, GB 5009.242–2017, and GB 5009.13–2017, respectively. Using a calibrated one-in-ten-thousand balance, 1 gram of processed sample (liver, heart, tibia ash) was weighed into a 50 mL conical bottle. The samples were digested using the wet digestion method, adding a mixture of nitric acid and perchloric acid (1:4) solution, 10 mL, and soaking them overnight. The conical bottle was slowly heated on an electric furnace, and the digested samples turned brown, then cooled down. After adding the mixed acid, the heating was continued, repeated several times until the digestion solution became transparent yellow-green and finally evaporated to approximately 2–3 mL. The digested samples were filtered, transferred without loss to a 50 mL volumetric flask, diluted with deionized water and mixed homogeneously. The content of zinc, iron, copper, and manganese was determined using a Shimadzu AA-7000 flame atomic absorption spectrophotometer, with wavelengths of 213.9 nm, 248.3 nm, 279.5 nm, and 324.8 nm, respectively. The detection limits for zinc, iron, copper, and manganese with the flame atomic absorption spectrophotometer system were 0.001–0.006 mg/L, 0.009–0.038 mg/L, 0.008–0.032 mg/L, and 0.006–0.023 mg/L, respectively.

### Statistical analysis

This study used SPSS 26.0 software (SPSS Inc., Chicago, Illinois, United States) for one-way ANOVA analysis, with treatment as a fixed effect, followed by a post-hoc Tukey procedure to analyze whether there was a significant effect between different treatments. Graphs were generated using GraphPad Prism 9.4 software. The significance level was set at *p* < 0.05.

## Results

### Growth performance

During three stages, namely 21–35 days, 36–50 days, and 21–50 days, there were no significant effects observed (*p* > 0.05; Table 3) on average daily gain (ADG), average daily feed intake (ADF), and feed conversion ratio (FI) when comparing the 1,000 mg/kg sulfate group (ITM) with the organic and inorganic mixed group (MIX) at doses of 200, 400, and 600 mg/kg.

**Table 3:** Effect of supplementation of ITM and MIXs in basal rations on growth performance of chickens.

Items	1,000 mg/kg ITM	200 mg /kg MIX	400 mg/kg MIX	600 mg/kg MIX	SEM	*p*-value
Body weight						
Day 21, g	438.54	438.54	438.54	438.54	0.52	1.00
Day 35, g	1060.07	1057.80	1086.22	1092.47	8.70	0.40
Day 50, g	1859.80	1850.97	1894.03	1874.03	21.97	0.91
Starter stage (day 21–35)						
ADG, g	41.44	41.28	43.18	43.60	0.57	0.38
ADFI, g	77.26	74.28	77.15	79.02	0.95	0.39
FCR	1.87	1.92	1.79	1.82	0.02	0.35
Finisher stage (day 36–50)						
ADG, g	53.32	52.55	53.86	52.10	1.08	0.94
ADFI, g	113.66	117.38	122.52	128.86	3.61	0.48
FCR	2.14	2.23	2.28	2.49	0.07	0.30
Total stage (day 21–50)						
ADG, g	47.38	47.07	48.52	47.85	0.73	0.91
ADFI, g	94.59	95.32	98.98	98.01	1.87	0.81
FCR	2.00	2.02	2.04	2.05	0.02	0.85

The 1,000 mg/kg ITM group was supplemented with 1,000 mg/kg of sulfate trace minerals in the basal diet; The 200 mg/kg MIX group was supplemented with mixed trace mineral elements (50% sulfate + 50% small pep-tide chelates); The 400 mg/kg MIX group was supplemented with mixed trace mineral elements (50% sulfate + 50% small pep-tide chelates); The 600 mg/kg MIX group was supplemented with mixed trace mineral elements (50% sulfate + 50% small pep-tide chelates). Numbers in the same row followed by different letters are statistically different (*p* < 0.05).

### Slaughter performance indicators

Based on the results shown in Table 4, there were no significant differences (*p* > 0.05; Table 4) observed in terms of carcass weight, dressing percentage, slaughter rate, breast muscle rate, and abdominal fat rate between the MIX groups at doses of 200, 400, and 600 mg/kg, and the 1,000 mg/kg ITM group. Among all treatment groups, the organic and inorganic mixed group at a dose of 600 mg/kg exhibited the highest breast muscle rate (*p* > 0.05; Table 4).

**Table 4:** Effect of supplementation of ITM and MIX in basal rations on slaughter performance of chickens.

Item	1,000 mg/kg ITM	200 mg/kg MIX	400 mg /kg MIX	600 mg/kg MIX	SEM	*p*-value
Dressing percentage	90.19	90.11	88.85	89.52	0.34	0.48
Semi-eviscerated weight, g	1415.60	1389.00	1405.50	1392.17	9.81	0.77
eviscerated weight, g	1211.40	1200.50	1233.17	1187.33	8.20	0.26
Breast muscle percentage	20.13	20.66	21.92	23.15	0.52	0.21
Leg muscle percentage	19.79	18.81	21.25	20.01	0.39	0.20
Abdominal fat percentage	2.86	3.33	3.25	3.26	0.15	0.71

The 1,000 mg/kg ITM group was supplemented with 1,000 mg/kg of sulfate trace minerals in the basal diet; The 200 mg/kg MIX group was supplemented with mixed trace mineral elements (50% sulfate + 50% small peptide chelates); The 400 mg/kg MIX group was supplemented with mixed trace mineral elements (50% sulfate + 50% small pep-tide chelates); The 600 mg/kg MIX group was supplemented with mixed trace mineral elements (50% sulfate + 50% small peptide chelates). Numbers in the same row followed by different letters are statistically different (*p* < 0.05).

### Serum biochemical indices

At day 35, compared to the 1,000 mg/kg ITM group, the levels of albumin, alkaline phosphatase, total cholesterol, total protein, and urea nitrogen in the serum showed no significant effects in the 200, 400, and 600 mg/kg MIX groups (*p* > 0.05; Table 4).

At day 50, the levels of cholesterol and urea nitrogen in the 1,000 mg/kg ITM group were significantly higher than those in the 200, 400, and 600 mg/kg MIX groups (*p* < 0.01; Table 4).

### Antioxidant indicators

At 35 days, the levels of Cu-Zn SOD in the serum and liver of the 400 and 600 mg/kg MIX groups were significantly increased compared to the 1,000 mg/kg ITM group (*p* < 0.05; Table 5), while there were no significant differences in other antioxidant indicators (*p*>0.05; Table 5).

**Table 5:** Effect of supplementation of ITM and MIX in basal rations on slaughter performance of chickens.

Item	1,000 mg/kg ITM	200 mg/kg MIX	400 mg /kg MIX	600 mg/kg MIX	SEM	*p*-value
35d						
Albumin, g/L	17.13	16.49	17.17	19.32	0.52	0.25
Alkaline phosphatase, King’s unit/100 mL	32.53	24.93	31.66	28	2.1	0.56
Total cholesterol, mmol/L	4.61	4.69	4.36	4.65	0.15	0.88
Urea nitrogen, mmol/L	8.90	9.03	8.90	7.87	0.25	0.32
Total protein, ug/mL	28.92	31.52	30.8	31.12	0.72	0.60
50d						
Albumin, g/L	12.32	13.7	13.25	14.19	0.34	0.28
Alkaline phosphatase, King’s unit/100 mL	21.46	21.55	24.27	36.51	2.25	0.17
Total cholesterol, mmol/L	3.1^a^	1.55^b^	1.50^b^	1.64^b^	0.12	<0.01
Urea nitrogen, mmol/L	7.2^a^	2.5^b^	1.58^b^	3.08^b^	0.26	<0.01
Total protein, ug/mL	23.81	27.06	22.84	29.11	1.09	0.17

The 1,000 mg/kg ITM group was supplemented with 1,000 mg/kg of sulfate trace minerals in the basal diet; The 200 mg/kg MIX group was supplemented with mixed trace mineral elements (50% sulfate + 50% small peptide chelates); The 400 mg/kg MIX group was supplemented with mixed trace mineral elements (50% sulfate + 50% small pep-tide chelates); The 600 mg/kg MIX group was supplemented with mixed trace mineral elements (50% sulfate + 50% small peptide chelates). Numbers in the same row followed by different letters are statistically different (*p* < 0.05).

At 50 days, compared to the 1,000 mg/kg ITM group, the levels of glutathione peroxidase in the serum of the 200, 400, and 600 mg/kg MIX groups were significantly in-creased, and the levels of MDA in the serum were significantly reduced (*p* < 0.01; Table 5), while there were no significant differences in other antioxidant indicators (*p*>0.05; Table 5).

### Mineral deposition in the liver

**Figure 1:**
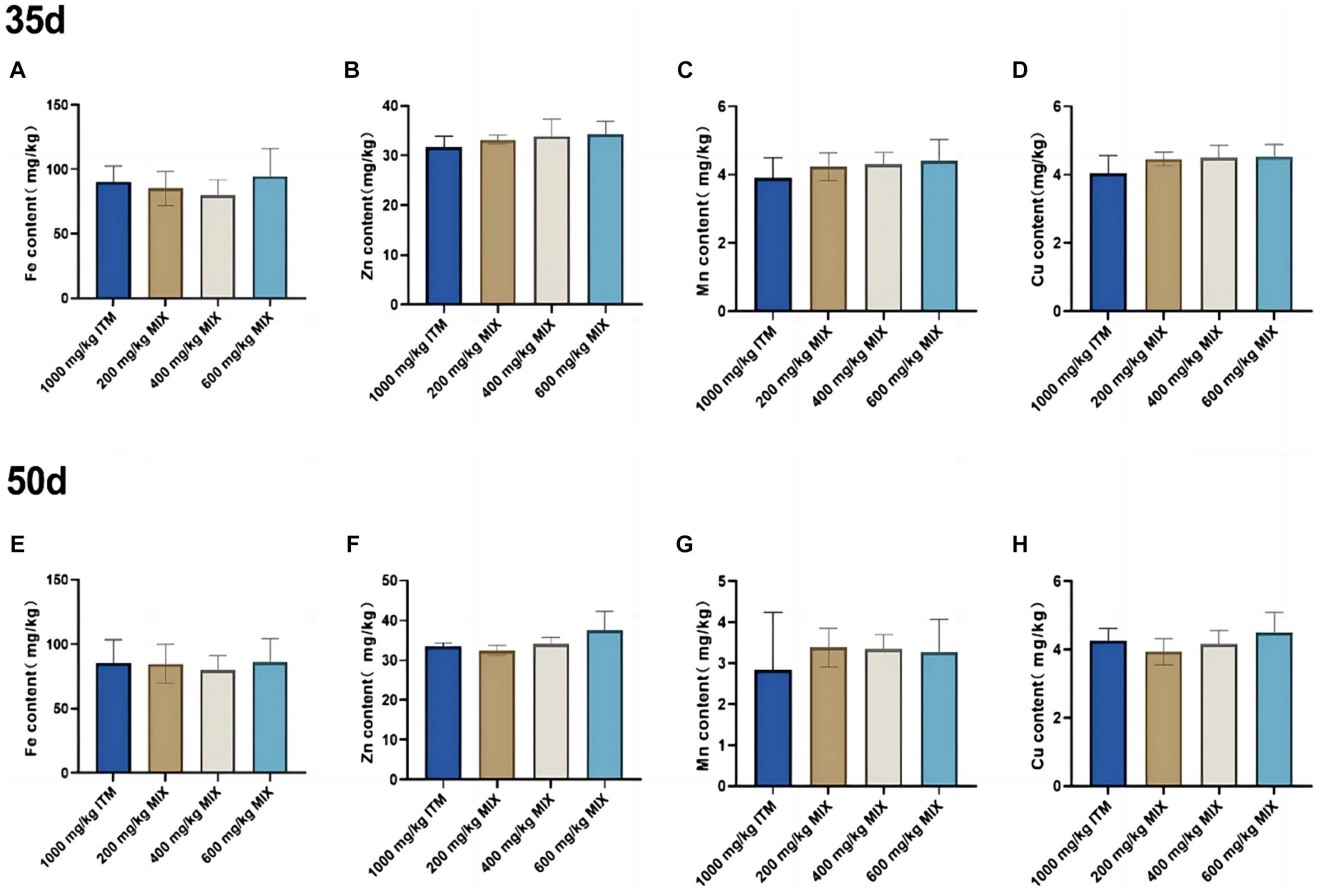
The influence of adding ITM and MIX to the basal diet on the deposition of trace elements in broiler chicken’s liver. **(A-D)** show the content of trace elements Fe, Zn, Mn, and Cu in the liver of broilers at 35 days. **(E-H)** show the content of trace elements Fe, Zn, Mn, and Cu in the liver of broilers at 50 days. The 1,000 mg/kg ITM group was supplemented with 1,000 mg/kg of sulfate trace minerals in the basal diet; The 200 mg/kg MIX group was supplemented with mixed trace mineral elements (50% sulfate +50% small pep-tide chelates); The 400 mg/kg MIX group was supplemented with mixed trace mineral elements (50% sulfate +50% small peptide chelates); The 600 mg/kg MIX group was supplemented with mixed trace mineral elements (50% sulfate +50% small peptide chelates). Significant deviations are denoted by asterisks (^*^p < 0.05, ^**^p < 0.01, ^***^p < 0.001).

At 35 and 50d, there were no significant differences (*p*>0.05; Figure 1) in the levels of iron, zinc, manganese, and copper elements in the liver between the 200, 400, and 600 mg/kg MIX group and the 1,000 mg/kg ITM group.

### Mineral deposition in the heart

**Figure 2:**
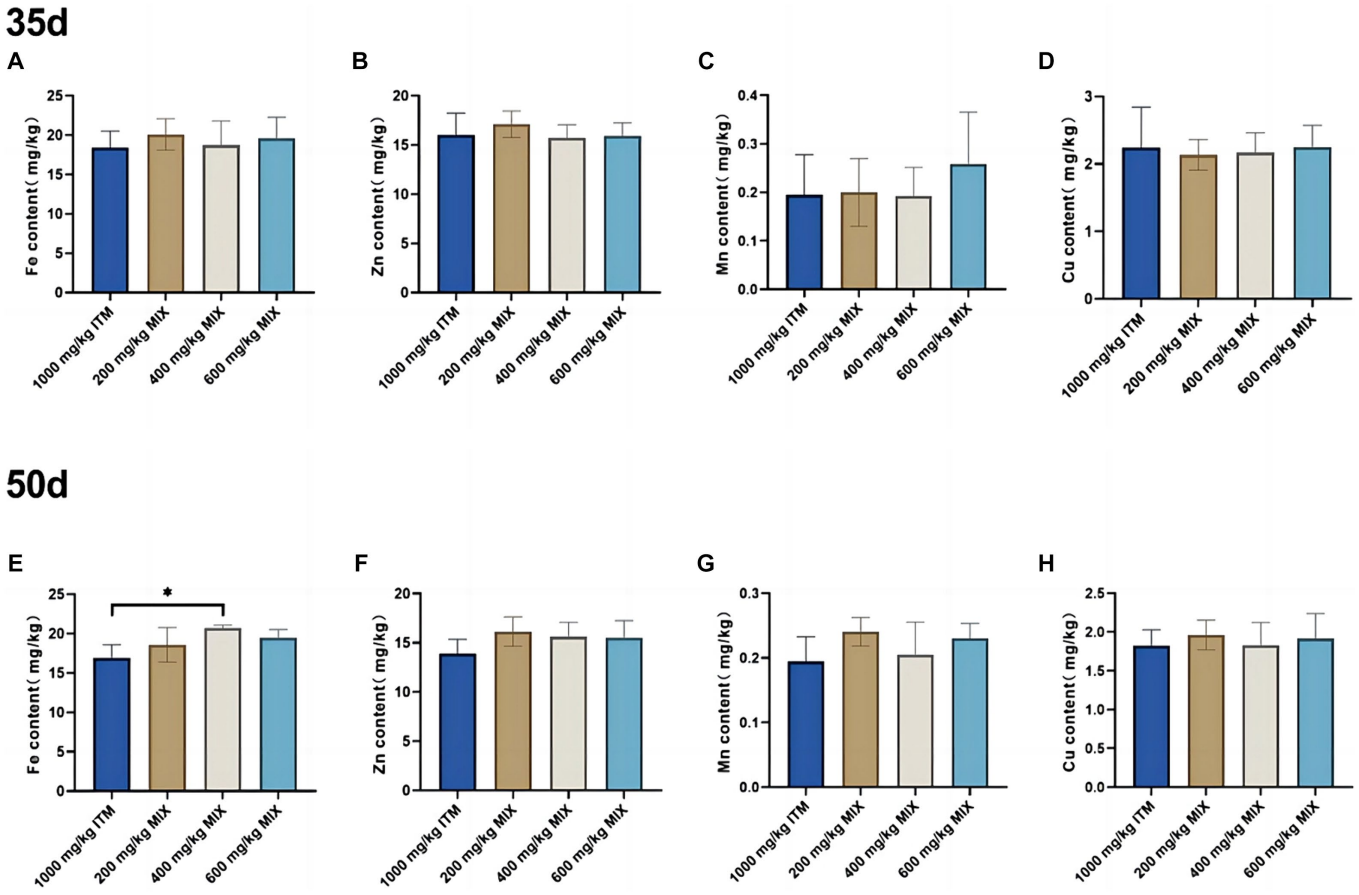
The influence of adding ITM and MIX to the basal diet on the deposition of trace elements in broiler chicken’s heart. **(A-D)** show the content of trace elements Fe, Zn, Mn, and Cu in the heart of broilers at 35 days. **(E-H)** show the content of trace elements Fe, Zn, Mn, and Cu in the heart of broilers at 50 days. The 1,000 mg/kg ITM group was supplemented with 1,000 mg/kg of sulfate trace minerals in the basal diet; The 200 mg/kg MIX group was supplemented with mixed trace mineral elements (50% sulfate +50% small pep-tide chelates); The 400 mg/kg MIX group was supplemented with mixed trace mineral elements (50% sulfate +50% small peptide chelates); The 600 mg/kg MIX group was supplemented with mixed trace mineral elements (50% sulfate +50% small peptide chelates). Significant deviations are denoted by asterisks (^*^p < 0.05, ^**^p < 0.01, ^***^p < 0.001).

At 35d, there were no significant differences (*p* > 0.05; Figure 2) in the iron, zinc, manganese, and copper elements in the heart between the 200, 400, and 600 mg/kg MIX groups and the 1,000 mg/kg ITM group. At 50d, in comparison with the 1,000 mg/kg ITM group, the iron element in the heart significantly increased (*p* < 0.05; Figure 2E) in the 600 mg/kg MIX group.

### Mineral deposition in the tibia bone

**Figure 3:**
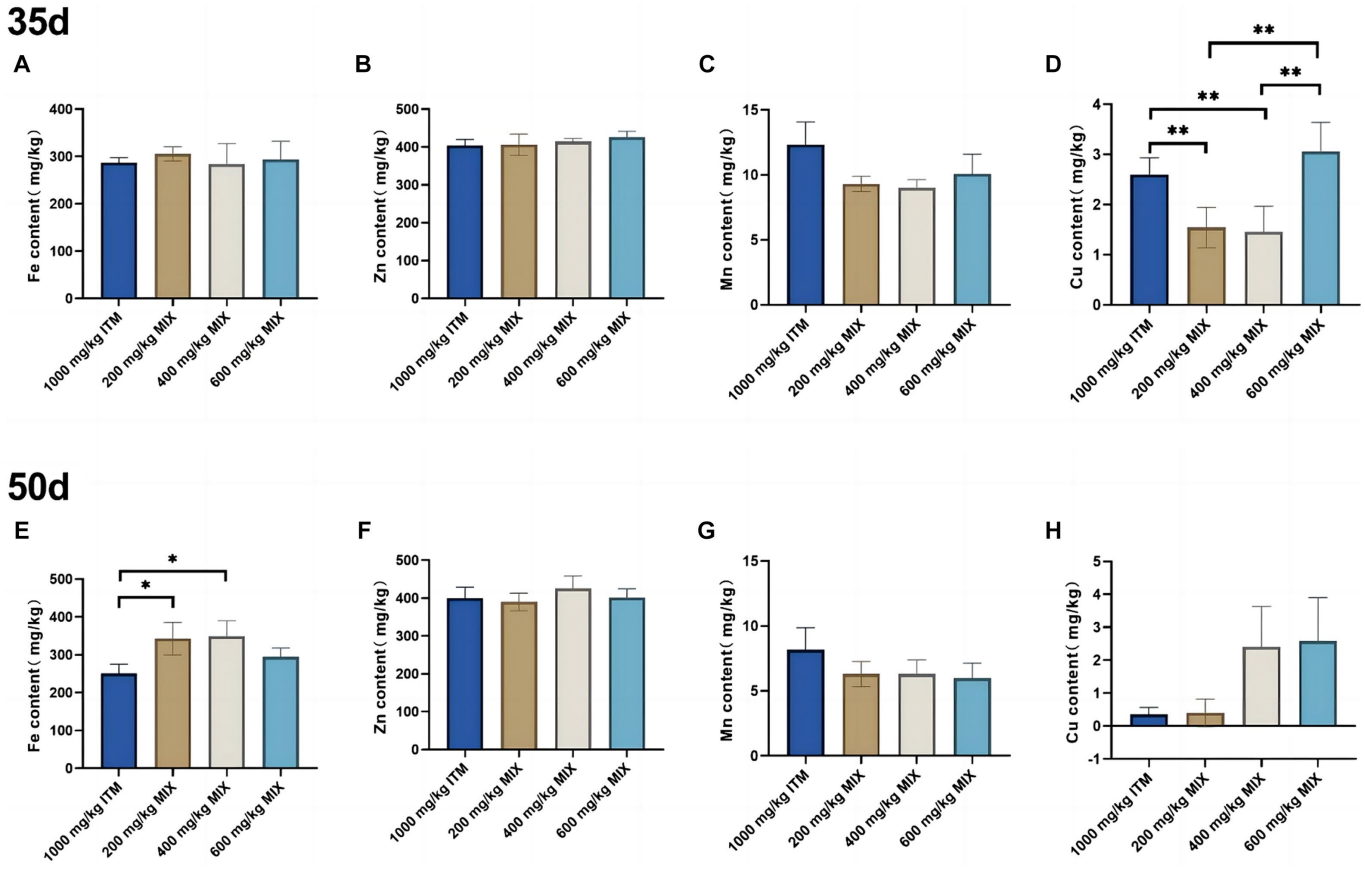
The influence of adding ITM and MIX to the basal diet on the deposition of trace elements in broiler chicken’s tibia. **(A-D)** show the content of trace elements Fe, Zn, Mn, and Cu in the tibias of broilers at 35 days. **(E-H)** show the content of trace elements Fe, Zn, Mn, and Cu in the tibias of broilers at 50 days. The 1,000 mg/kg ITM group was supplemented with 1,000 mg/kg of sulfate trace minerals in the basal diet; The 200 mg/kg MIX group was supplemented with mixed trace mineral elements (50% sulfate +50% small pep-tide chelates); The 400 mg/kg MIX group was supplemented with mixed trace mineral elements (50% sulfate +50% small peptide chelates); The 600 mg/kg MIX group was supplemented with mixed trace mineral elements (50% sulfate +50% small peptide chelates). Significant deviations are denoted by asterisks (^*^p < 0.05, ^**^p < 0.01, ^***^p < 0.001).

At 35d and 50d, compared with the 1,000 mg/kg ITM group, the copper element in the tibia significantly decreased (*p* < 0.01; Figure 3D) in the 200 and 400 mg/kg MIX groups. At 50d, compared with the 1,000 mg/kg ITM group, the iron element in the tibia significantly decreased (*p* < 0.05; Figure 3E) in the 200 and 400 mg/kg MIX groups.

### Mineral content in feces

**Figure 4:**
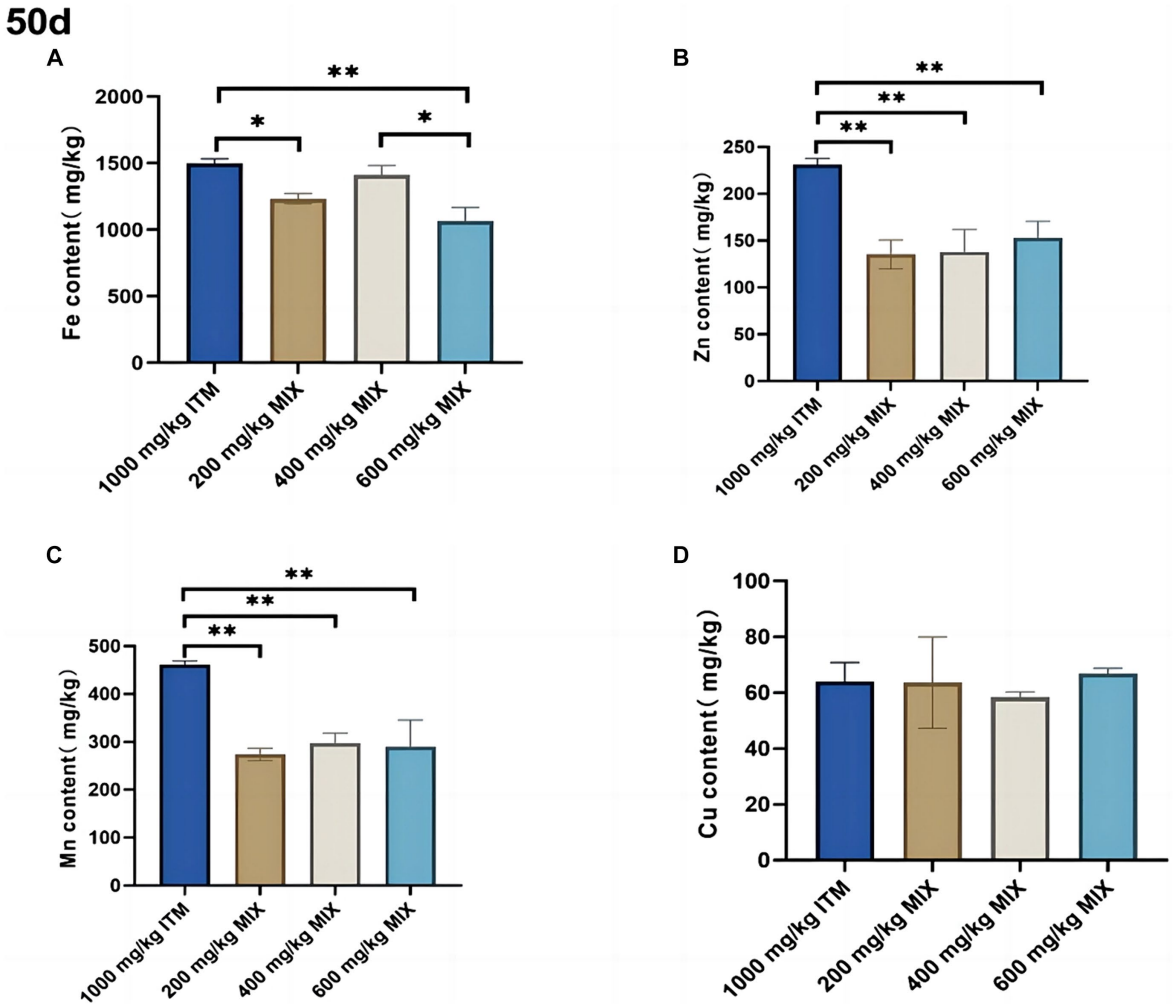
The influence of adding ITM and MIX to the basal diet on the deposition of trace elements in broiler chicken’s feces. **(A-D)** show the content of trace elements Fe, Zn, Mn, and Cu in the feces of broilers at 50 days. The 1,000 mg/kg ITM group was supplemented with 1,000 mg/kg of sulfate trace minerals in the basal diet; The 200 mg/kg MIX group was supplemented with mixed trace mineral elements (50% sulfate +50% small pep-tide chelates); The 400 mg/kg MIX group was supplemented with mixed trace mineral elements (50% sulfate +50% small peptide chelates); The 600 mg/kg MIX group was supplemented with mixed trace mineral elements (50% sulfate +50% small peptide chelates). Significant deviations are denoted by asterisks (^*^p < 0.05, ^**^p < 0.01, ^***^p < 0.001).

At 50d, compared with the 1,000 mg/kg ITM group, the zinc and manganese elements in the feces significantly decreased (*p* < 0.01; Figures 4B,C) in the 200, 400, and 600 mg/kg MIX groups. Compared with the 1,000 mg/kg ITM group, the iron element in the feces significantly decreased (*p* < 0.01; Figure 4A) in the 200 and 600 mg/kg MIX groups (see Table 6).

**Table 6:** Effect of supplementation of ITM and MIXs in basal rations on serum and liver antioxidant parameters of chickens.

Item	1,000 mg/kg ITM	200 mg/kg MIX	400 mg /kg MIX	600 mg/kg MIX	SEM	*p*-value
35d						
Serum antioxidant parameters						
MDA, nmol/mL	3.18	2.63	3.06	2.63	0.1	0.21
T-AOC, U/mL	0.62	0.62	0.63	0.58	0.01	0.65
Cu/Zn SOD, U/mL	186.77^b^	190.3^ab^	209.29^a^	209.09^a^	3.21	< 0.05
GSH-Px, U/mL	1458.28	1548.87	1527.81	1533.38	26.8	0.66
Liver antioxidant parameters						
MDA, nmol/mgprot	0.55	0.46	0.47	0.55	0.02	0.20
T-AOC, mmol/mgprot	0.05	0.06	0.06	0.06	0.00	0.17
Cu/ZnSOD, U/mgprot	296.86^b^	293.53^b^	342.20^a^	327.28^a^	3.11	<0.01
GSH-Px, U/mg	49.84	52.37	53,87	52.58	0.84	0.43
50d						
Serum antioxidant parameters						
MDA, nmol/mL	6.47^a^	2.48^b^	2.9^b^	2.57^b^	0.08	<0.01
T-AOC, U/mL	0.52	0.5	0.58	0.54	0.01	0.19
Cu/Zn SOD, U/mL	196.93	175.07	181.57	178.9	2.56	0.08
GSH-Px, U/mL	1260.12^b^	1681.61^a^	1811.47^a^	1769.32^a^	30.54	<0.01
Liver antioxidant parameters						
MDA, nmol/mgprot	0.3	0.36	0.33	0.28	0.02	0.37
T-AOC, mmol/mgprot	0.04	0.04	0.05	0.04	0.00	0.05
Cu/ZnSOD, U/mgprot	212.93	232.33	263.62	250.46	5.75	0.03
GSH-Px, U/mg	39.58	41.64	44.16	42.79	1.06	0.52

The 1,000 mg/kg ITM group was supplemented with 1,000 mg/kg of sulfate trace minerals in the basal diet; The 200 mg/kg MIX group was supplemented with mixed trace mineral elements (50% sulfate + 50% small peptide chelates); The 400 mg/kg MIX group was supplemented with mixed trace mineral elements (50% sulfate + 50% small pep-tide chelates); The 600 mg/kg MIX group was supplemented with mixed trace mineral elements (50% sulfate + 50% small peptide chelates). Numbers in the same row followed by different letters are statistically different (*p* < 0.05).

## Discussion

A common method for evaluating the growth performance of broiler chickens is by using growth parameters. The results of this experiment showed that compared to the group supplemented with 1,000 mg/kg of sulfate, the group supplemented with a mixture of organic and inorganic trace elements at levels of 200, 400, and 600 mg/kg did not negatively affect the growth performance of broiler chickens. Advanced chelation technology can reduce the supplementation of trace elements for young chickens, as chelates have higher bioavailability of trace elements. The findings of this study are consistent with the views of Marco et al. (18), who suggest that reducing the supplementation of copper, iron, zinc, and manganese (to 50% of the commercial recommended dosage) in the form of glycine metal chelates or amino acid metal chelates does not significantly impair the growth performance of poultry. Furthermore, Zhu et al. (19) found that substituting inorganic trace minerals with chelated trace minerals at levels 30 and 50% lower than the inorganic mineral level had no effect on the growth performance of broiler chickens. In the Kong et al. (16) study, it was found that the use of low-dose small-peptide chelated minerals can completely replace inorganic trace mineral supplements without any negative impact on the production performance of broilers. This is consistent with our research findings. However, adopting a combination of inorganic and organic forms can reduce the amount of small peptide chelated minerals used and subsequently lower costs. Although several studies suggest that substitution of inorganic trace minerals with lower levels of organic trace minerals does not have a detrimental effect on birds, our results differ from those of some studies. The study by Sirri et al. (20) found that adding organic trace minerals significantly improved weight and feed conversion ratio (FCR) in broiler chickens at 51 days of age. Singh et al. (21) also improved weight and optimized feed conversion ratio to some extent by supplementing broiler chickens with copper, iron, manganese, and zinc in the form of methionine chelates or yeast proteins. The different results of these studies may be attributed to variations in the source and dosage of supplemented trace minerals. In conclusion, the results of this experiment demonstrate that replacing high levels of inorganic trace minerals with a lower level mixture of organic and inorganic trace elements can meet the requirements of broiler chickens for trace minerals without adversely affecting their growth performance.

Slaughter performance can intuitively reflect the level of meat production rates, which is an important indicator for evaluating the economic value and meat yield of animals. Based on the results of this study, the meat production performance of the experimental chickens was evaluated. It was found in this study that compared to the inorganic additive group, the addition of organic and inorganic mixed trace elements at levels of 200, 400, and 600 mg/kg in the feed had no significant effects on the slaughter rate, semi-dressed carcass weight, dressed carcass weight, breast muscle percentage, leg muscle percentage, and abdominal fat percentage. Similar to the results of this study, Sirri et al. (20) found in their research that supplementing organic trace minerals does not affect carcass characteristics. However, other studies have reported different results regarding the effects of adding organic trace minerals on carcass characteristics. In a study by Zhao et al. (22), it was found that compared to 100% inorganic additives, the addition of a mixture of inorganic and chelated trace minerals (50:50) in the feed increased breast muscle yield in male broiler chickens (Cobb 700), but had no effect on the carcass characteristics of 52-day-old broiler chickens (ROSS 308). Interestingly, this study also found that the addition of 600 mg/kg of organic and inorganic mixed trace elements resulted in a breast muscle percentage of 23.15%, which was the highest but not significantly different from the other treatment groups. This suggests a positive impact of 600 mg/kg of organic and inorganic mixed trace minerals in improving breast muscle yield.

Serum biochemical indicators are key indicators reflecting the nutritional status of animals (23). In the animal body, manganese plays an important role in the synthesis of steroid hormones and is involved in cholesterol metabolism (24). Zinc is involved in bone formation, immune response mediated by cells, host defense system, sexual maturity, tissue growth, and also participates in lipid metabolism (25, 26). Fatma et al. (27) found that zinc can lower serum cholesterol levels when it is added at low concentrations. Our study found that feeding broiler chickens with a low concentration of mixed organic and inorganic trace elements is more effective in reducing total cholesterol levels than providing only inorganic trace minerals. Having a high level of blood urea nitrogen is an indication of disturbed protein metabolism and is considered as an important biomarker for renal health of broilers (28). In our study, adding a mixture of organic and inorganic trace elements to the feed significantly reduced uric acid levels in chickens, indicating a beneficial effect of adding organic trace elements on kidney function.

Excessive free radicals in the animal body cause damage to cells, which in turn leads to damage to the organism, resulting in oxidative stress. MDA is produced after the peroxidation of polyunsaturated fatty acids and can serve as a good marker for lipid peroxidation (29). The antioxidant enzyme system is primarily composed of GSH-Px and Cu-Zn SOD. They act as enzyme catalysts to scavenge free radicals, thereby protecting cells from the detrimental effects of the destruction process and alleviating oxidative stress in a timely manner. This study found that low concentrations of organic and inorganic mixed trace elements significantly increased the content of Cu/Zn SOD in the serum and liver, and decreased the level of MDA in the serum. Similar to the findings of this study, Umar Yaqoob et al. (30) studied the replacement of inorganic trace minerals with composite glycine salts and found that the total antioxidant capacity of the liver in the treatment groups with 70 and 50% composite glycine salts was higher than that in the inorganic group and the organic and inorganic mixed group. Furthermore, Wang et al. (29) found that the addition of 50 and 62.5% commercially available organic minerals significantly reduced the levels of MDA, providing better alleviation of lipid peroxidation. Similar results were also obtained in the study conducted by Aksu et al. (31). In the Kong et al. (16) study, it was found that the use of low-dose small peptide chelated minerals can completely replace inorganic trace mineral supplements, which significantly improves the liver’s antioxidant capacity in broilers, consistent with our research results. Overall, the three organic and inorganic mixed groups showed superior antioxidant capacity compared to the inorganic group.

Poultry ingest feed, and after that, trace minerals pass through the intestinal epithelial cells and enter the bloodstream via the digestive tract. These trace minerals are primarily deposited in organs such as the liver, while the remainder is transferred to the bones, with a small portion deposited in tissues such as muscles and the brain. The deposition of trace minerals in the poultry’s tissues partially reflects their bioavailability. Among them, the liver, chicken breast, and tibia are particularly sensitive to the deposition of trace minerals (32, 33). Wang et al. (34) found no significant differences in the concentrations of copper, zinc, and manganese in the liver between the groups supplemented with 37.5, 50, and 62.5% organic trace minerals (OTM) and the inorganic group, indicating that the source and level of minerals did not affect the deposition of minerals in the liver. Additionally, Mondal et al. (35) found higher concentrations of trace elements (copper, iron, manganese, and zinc) in serum, liver, and muscle samples in the treatment group supplemented with chelated trace minerals. This suggests that the specific form of supplementation enhances the absorption capacity of trace minerals. This study also observed no significant differences in the concentrations of iron, copper, zinc, and manganese in the liver and heart between the inorganic group and the organic–inorganic mixed treatment group. Compared to the organic group, the low-concentration organic–inorganic mixed group showed an upward trend in the concentrations of iron, copper, zinc, and manganese in the liver and heart, suggesting that small peptide chelators may have a promoting effect on these minerals.

Skeletons are complex heterogeneous tissues that play a major role in animal growth (36). Copper, iron, manganese, and zinc are essential minerals for maintaining the healthy growth and development of the skeleton (37). The zinc concentration in the tibia is an important indicator for predicting good growth in poultry. Zinc serves as a co-factor for various bone mineralization enzymes, and zinc deficiency in broiler chickens can lead to shortened and thickened tibias, directly affecting the growth status of poultry and the economic income of farmers (38). According to this study, the organic zinc group was supplemented with an adequate amount of zinc, and there was no significant difference in the zinc concentration in the tibias of the other organic and inorganic mixed zinc groups with lower supplementation levels, further demonstrating that low-dose organic zinc does not have a negative impact on the growth of broiler chickens. In Ma et al. (39), the experimental group supplemented with different levels of glycine-chelated iron significantly increased the iron concentration in the tibias. Iron concentrations in tibias did not differ significantly after 35 days, but at 50 days, the organic and inorganic mixed zinc groups with 200 mg/kg and 400 mg/kg supplementation significantly increased the concentration of iron in the tibias, indicating that small peptide chelators may promote tibial iron and zinc. Studies have shown that compared to other trace minerals, the manganese content in the tibias of poultry seems to be more susceptible to the source and supplementation level of trace minerals (40). In this study, at 35 days, there was no significant difference in the manganese concentration in the tibias between the 1,000 mg/kg sulfate manganese group and the three organic and inorganic mixed manganese groups, indicating that supplementation with low levels of organic manganese does not affect the manganese concentration in the tibias of growing chicken.

Mineral excretion has the potential to pollute the environment and waste resources. By controlling the dietary intake of trace elements, the excessive emission of heavy metals in the environment can be reduced. This study found that the organic–inorganic mixed treatment group significantly decreased the emission of zinc and manganese. Additionally, compared to the inorganic sulfate group, the organic–inorganic mixed group at doses of 200 mg/kg and 600 mg/kg also reduced the excretion of iron in feces. Similar to the findings of this study, Lippens et al. (41) found that reducing the levels of trace minerals in broiler feed, such as 2.5 ppm Bioplex Cu, 10 ppm Bioplex Zn, Bioplex Fe, and Bioplex Mn, had no negative effects on broiler growth performance and significantly reduced the excretion of trace minerals. Bao et al. (42) in a cage rearing experiment with Cobb broilers, did not find any promotion of broiler growth when feeding a high level of organic chelated trace minerals, and the results of fecal excretion concentration measurement showed no reduction in the excretion of copper, zinc, and manganese. Therefore, supplementing animals with high levels of organic minerals in the diet may lead to overload, posing a potential threat to environmental pollution and resource wastage. By controlling the dietary intake of trace nutrients, the excessive accumulation of heavy metals in agricultural fields can be reduced. There is no doubt that this strategy is an effective and feasible solution for reducing mineral pollution. In our study, the addition of organic chelators and inorganic salts in the mixed treatment group significantly reduced mineral content without affecting growth performance. This may be due to the presence of a unique transport system that allows minerals to pass efficiently through the intestinal wall in the form of chelates, resulting in higher biological utilization. Our research aligns with previous studies, suggesting that supplementation with low doses of organic forms not only has no adverse effects on growth performance, but also effectively mitigates environmental pressure (43).

## Conclusion

In this study, organic and inorganic mineral mixtures at concentrations of 200, 400, and 600 mg/kg were used as substitutes for inorganic trace mineral supplements, showing no adverse effects on the productivity of 817 broilers. This substitution method could reduce serum cholesterol and urea nitrogen levels, improve the activity of antioxidant indicators in serum and liver, promote iron deposition in the tibia, significantly decrease the mineral content in feces. Among them, 400 mg/kg was more effective in promoting iron deposition in the tibia and reducing the mineral content in feces.

## Data availability statement

The raw data supporting the conclusions of this article will be made available by the authors, without undue reservation.

## Ethics statement

The study was approved by the Ethical Committee and conducted under the supervision of the Institutional Animal Care and Use Committee of Foshan University (ApprovalID:FOSU#19-025). The study was conducted in accordance with the local legislation and institutional requirements.

## Author contributions

XH: Writing – original draft, Writing – review & editing. JK: Writing – original draft, Writing – review & editing. CZ: Writing – review & editing. XY: Writing – review & editing. TQ: Writing – review & editing. ZC: Writing – review & editing. HZ: Writing – review & editing.
